# Reversion of the Inflammatory Markers in Patients With Chronic Limb‐Threatening Ischemia

**DOI:** 10.1161/JAHA.123.031922

**Published:** 2024-04-12

**Authors:** Joana Ferreira, Susana Roque, Alexandre Lima Carneiro, Adhemar Longatto‐Filho, Isabel Vila, Cristina Cunha, Cristina Silva, Amílcar Mesquita, Jorge Cotter, Margarida Correia‐Neves, Armando Mansilha, Pedro Guimarães Cunha

**Affiliations:** ^1^ Vascular Surgery Department—Physiology and Surgery Centro Hospitalar Universitário de São João Porto Portugal; ^2^ Life and Health Science Research Institute (ICVS), School of Medicine University of Minho Braga Portugal; ^3^ ICVS/3B’s—PT Government Associated Laboratory Braga Portugal; ^4^ Academic Center Hospital da Senhora da Oliveira Guimarães Portugal; ^5^ Clinical Academic Center Hospital de Trás‐os‐Montes e Alto Douro—Professor Doutor Nuno Grande—CACTMAD Vila Real Portugal; ^6^ Radiology Department ULSAM Viana do Castelo Portugal; ^7^ Department of Pathology (LIM‐14) University of São Paulo School of Medicine São Paulo Brazil; ^8^ Molecular Oncology Research Center Barretos Cancer Hospital Barretos São Paulo Brazil; ^9^ Center for the Research and Treatment of Arterial Hypertension and Cardiovascular Risk, Hospital da Senhora da Oliveira Guimarães Portugal; ^10^ Internal Medicine Department Hospital da Senhora da Oliveira, Guimarães Braga Portugal; ^11^ Vascular Surgery Department—Hospital da Senhora da Oliveira Guimarães Portugal; ^12^ Department of Angiology and Vascular Surgery Centro Hospitalar Universitário de São João Porto Portugal; ^13^ Faculty of Medicine University of Porto Porto Portugal

**Keywords:** atherosclerosis, chronic limb‐threatening ischemia, inflammation, peripheral artery disease, Vascular Disease

## Abstract

**Background:**

Peripheral artery disease is characterized by an intense inflammatory process that can be associated with a higher mortality rate, particularly in chronic limb‐threatening ischemia (CLTI). This study aims to compare the evolution of inflammatory markers between patients with claudication with those with CLTI at 3, 6, and 12 months.

**Methods and Results:**

An observational, single‐center, and prospective study was conducted. A total of 119 patients with peripheral artery disease (65 with claudication and 54 with CLTI) were observed and inflammatory markers collected at admission and 3, 6, and 12 months. At admission, patients with CLTI, when compared with patients with claudication, had significantly higher serum levels of C‐reactive protein and fibrinogen (positive acute‐phase proteins) and lower serum level of albumin, total cholesterol, and high‐density lipoprotein (negative acute‐phase proteins): C‐reactive protein (g/dL), 2.90 (25th–75th percentile, 2.90–4.90) versus 6.80 (25th–75th percentile, 2.90–53.26) (*P*=0.000); fibrinogen (mg/dL), 293.00 (25th–75th percentile, 269.25–349.00) versus 415.50 (25th–75th percentile, 312.00–615.75) (*P*=0.000); total cholesterol (mg/dL), 161.79±95% [152.74–170.85] versus 146.42%±95% [135.30–157.53] (*P*=0.034); high‐density lipoprotein (mg/dL), 50.00 (25th–75th percentile, 41.00–60.00) versus 37.00 (25th–75th percentile, 30.00–45.50) (*P*=0.000); albumin (g/dL): 4.00 (25th–75th percentile, 3.70–4.20) versus 3.60 (25th–75th percentile, 3.10–4.00) (*P*=0.003). The association between CLTI and total cholesterol was lost after adjusting for confounders. Three months after the resolution of the CLTI, there was an increase in the levels of negative acute‐phase proteins and a decrease in positive acute‐phase proteins. These inflammatory proteins did not register an evolution in patients with claudication. The differences in the inflammatory proteins between groups disappeared at 6 months.

**Conclusions:**

CLTI has an inflammatory environment that can be partially reverted after resolution of the ischemic process, emphasizing the importance of timely intervention.

**Nonstandard Abbreviations and Acronyms**
CLTI: chronic limb‐threatening ischemia

**Clinical Perspective**

**What Is New?**

- Patients with chronic limb‐threatening ischemia have serum inflammatory markers, mirroring a serious medical inflammatory condition, with potential deleterious impact.

**What Are the Clinical Implications?**

- This is one of the few papers showing that this inflammatory environment can be partially reverted with the resolution of the chronic limb‐threatening ischemia, underscoring the importance of a prompt intervention.

Lower extremity peripheral artery disease (PAD) is characterized by atherosclerosis occurring in the lower limbs and affects >230 million adults worldwide.^1^, ^2^ Due to its high prevalence in low‐ and middle‐income countries, PAD is becoming an increasingly global problem. The most advanced stage of PAD is chronic limb‐threatening ischemia (CLTI), which accounts for 11% of diagnosed PAD cases.^1^ Patients with CLTI have a worse prognosis, with at least 20% of them dying within 1 year of diagnosis.^3^

PAD and inflammation are particularly connected. Inflammation plays a significant role in the initiation and progression of atherosclerosis, particularly in PAD.^4^, ^5^ Traditional cardiovascular risk factors exert their proatherogenic role, at least in part, through an inflammatory mechanism.^4^, ^6^ Also important is to acknowledge the important role of inflammation on the acceleration of the systemic arteriosclerotic process, leading equally to terminal cardiovascular and renal disease.^7^, ^8^ Proinflammatory cytokines (interleukin‐6, tumor‐necrosis factor‐α, and tumor‐necrosis factor‐α soluble receptor‐II) are strongly linked to the prevalence of PAD.^6^ The lower limbs, which possess a large vascular bed, frequently harbor inflamed plaques that release inflammatory mediators. These mediators contribute to the development of coronary artery disease, thereby explaining the higher incidence of coronary artery disease in patients with PAD.^4^ The prevalence of coronary artery disease in PAD ranges from 43% to 90%, while the prevalence of PAD in coronary artery disease patients is <25%.^4^ Studies have shown that the severity of coronary atherosclerosis is related to the degree of inflammatory response in the affected limb.^4^ Consequently, it has been suggested that it is not PAD itself, but rather its systemic inflammatory activity, that is associated with an increased number of coronary events.^4^

The main aim of this study is to determine the evolution of the inflammatory parameters at 3, 6, and 12 months in patients with claudication and with CLTI.

The second objective is to assess the differences in inflammatory parameters over time between patients with claudication and those with CLTI.

## Methods

### Study Type, Inclusion/Exclusion Criteria, and Ethical Considerations

An observational, prospective study was conducted from January 2018 to July 2020 at a single institution. The COVID‐19 pandemic significantly affected the research work. Consecutive patients with PAD attending the vascular surgery consultations or admitted at the vascular surgery ward who fulfilled the required criteria were included. The inclusion criterion was PAD suggested by the clinical history and objective examination and confirmed with ankle–brachial index. The exclusion criterion was any disease responsible for body composition changes or proinflammatory state in the past 3 months.

Ethics approval was obtained from the local hospital, with the protocol number 75/2017. All the participants signed the informed consent.

The data that support the findings of this study are available from the corresponding author upon reasonable request.

### Clinical Characteristics

Patient's age, sex, PAD clinical stage (CLTI and claudication), arterial hypertension, diabetes, dyslipidemia, smoking habits, and medication were collected at admission and defined as stated previous state.^9^ Fontaine stage III was defined as persistent rest pain for >2 weeks.^10^ Fontaine stage IV was defined as ischemic skin lesions.^10^ Both stages were confirmed with the following hemodynamic parameters: ankle pressure <50 mm Hg, absent palpable ankle pulses, or toe pressure <30 mm Hg in patients with diabetes and incompressible vessels.^11^

The patients were scheduled for an evaluation at 3, 6, and 12 months. The clinical evolution, amputation rate (major and minor), death, and causes of death were registered.

### Inflammatory Parameters

The serum inflammatory parameters determined were positive acute‐phase proteins (CRP [C‐reactive protein] and fibrinogen), negative acute‐phase proteins (albumin, total cholesterol, and high‐density lipoprotein [HDL]).

Blood samples were collected after a 10‐ to 12‐hour fast in the morning, taken into the appropriate Vacutainer, centrifuged within 5 minutes for 4000 cycles/min, and the serum was separated. The serum inflammatory parameters were evaluated at admission and at 3, 6, and 12 months of follow‐up, and tests were performed by routine procedures in the department of clinical chemistry.

### Statistical Analysis

Continuous variables were expressed as the mean with 95% CI or median and range. Between‐group differences in continuous variables were assessed with Student's *t* test or with the Mann–Whitney *U* test. Categorical variables were expressed as percentage. Between‐group comparisons for categorical variables were conducted using the χ^2^ test and the results expressed as odds ratio presented along with 95% CI. Multiple linear regression was used to adjust for potential confounding variables.

Repeated‐measure analysis was employed to study the evolution of acute‐phase proteins comparing 4 time points (admission, 3 months, 6 months, and 12 months).

A *P* value of <0.05 was considered significant. Statistical evaluation was performed using SPSS software version 20.0 (SPSS Inc., Chicago, IL).

To determine the differences in the inflammatory proteins between patients with claudication and patients with CLTI, a minimum sample of 53 patients for each group was necessary (with a significant level of 5% and for a power of 80%), calculated with G*Power 3.1.

## Results

### Clinical Characteristics at Admission

A total of 119 patients (95 men) with PAD with an average age of 67.58±9.60 years were enrolled in the study. The group of patients was evenly distributed, as 65 patients (54.62%) had claudication and 54 (45.38%) had CLTI. Nineteen of these patients had rest pain.

No differences were registered between patients with claudication and CLTI on age, sex, cardiovascular risk factors, and medication, except on smoking habits (Table 1). There was a significantly higher prevalence of smokers and a higher smoking load in the claudication group (claudication: median, 40.00 [25th–75th percentile, 15.00–60.00]; CLTI: median, 13.00 [25th–75th percentile, 00.00–40.00]; *P*=0.031; Table 1).

**Table 1:** Cardiovascular Risk Factor and Comorbidities of the PAD Population

	Claudication (n=65)	CLTI (n=54)	OR	95% CI	*P* value
Male, n (%)	55 (84.61)	40 (74.07)	1.79	0.71–4.48	0.216
Hypertension, n (%)	49 (75.38)	37 (68.52)	1.41	0.63–3.15	0.406
Dyslipidemia, n (%)	41 (63.08)	32 (59.26)	0.85	0.41–1.79	0.670
Ever smoker, n (%)	53 (81.53)	30 (55.56)	3.24	1.41–7.46	0.006*
Diabetes, n (%)	23 (35.38)	28 (51.85)	0.51	0.24–1.06	0.072
Coronary artery disease, n (%)	9 (13.85)	6 (11.11)	1.29	0.43–3.87	0.655
Statins, n (%)	58 (89.23)	44 (81.48)	1.70	0.59–4.90	0.331
Fibrate, n (%)	4 (6.15)	5 (9.26)	0.63	0.16–2.47	0.503
Ezetimibe, n (%)	3 (4.615)	3; 5.55	0.82	0.16–4.24	0.813
Antiplatelet, n (%)	54 (83.08)	40 (74.07)	1.43	0.59–3.48	0.424
ACEi/ARA, n (%)	20 (30.77)	16 (29.63)	0.99	0.45–2.20	0.989

ACEi indicates angiotensin‐converting enzyme inhibitor; ARA, angiotensin receptor antagonist; CLTI, chronic limb‐threatening ischemia; OR, odds ratio; and PAD, peripheral artery disease.

* *P*<0.005.

### Clinical Evolution

A total of 78.15% of patients attended the first 2 appointments, and 43.70% attended the third appointment; 23.53% were observed until the end of the study. Of the 65 patients with claudication, 14 (21.54%) improved their symptoms with medical therapy, 5 (7.69%) had worsening symptoms, and 1 was readmitted due to CLTI. Additionally, 13 patients discontinued their participation in the study, and 6 (5.04%) deaths were registered: 5 patients with CLTI, 1 patient with claudication. The causes of death were acute myocardial infarction (3 patients), heart failure (2 patients), pulmonary infection (1 patient), and lung cancer (1 patient). Thirty patients (55.56%) with CLTI were submitted to amputation (23 [42.59%] to minor amputation and 12 [22.22%] to major amputations). Of these, 29 patients underwent amputation within the initial 3 months, and 1 patient underwent amputation between the third and sixth months.

### Inflammatory Parameters

At admission, patients with CLTI had significantly higher serum levels of positive acute‐phase proteins (CRP and fibrinogen) and lower serum level of negative acute‐phase proteins (albumin, total cholesterol, and HDL), when compared with patients with claudication (Table 2).

**Table 2:** Positive (CRP and Fibrinogen) and Negative (HDL, Albumin, and Total Cholesterol) Acute‐Phase Proteins Determined in Patients With PAD at Admission

	Claudication (n=65)		CLTI (n=54)		*P* value
	Median	25th–75th percentile	Median	25th–75th percentile	
CRP, g/dL	2.90	2.90–4.90	6.80	2.90–53.26	0.000*
Fibrinogen, mg/dL	293.00	269.25–349.00	415.50	312.00–615.75	0.000*
HDL, mg/dL	50.00	41.00–60.00	37.00	30.00–45.50	0.000*
Albumin, g/dL	4.00	3.70–4.20	3.60	3.10–4.00	0.003*
Total cholesterol, mg/dL	161.79±95 (152.74–170.85)		146.42±95 (135.30–157.53)		0.034*

CLTI indicates chronic limb‐threatening ischemia; CRP, C‐reactive protein; HDL, high‐density lipoprotein; and PAD, peripheral artery disease.

* *P*<0.005.

Multiple linear regression analyses revealed that the presence of CLTI and smoking load were both predictors of CRP, with CLTI emerging as the stronger predictor (β=0.517, t=5.75, *P*=0.005; and β=0.258, t=2.87, *P*=0.005). Additionally, CLTI was identified as a predictor of fibrinogen, while smoking habits and aging were not significant contributors (β=0.447, t=4.93, *P*=0.000; β=0.25, t=0.26, *P*=0.793; and β=−0.023, t=0.23, *P*=0.821). Furthermore, CLTI and triglycerides were inversely associated with HDL (β=−0.363, t=− 4.20, *P*=0.000; and β=−0.290, t=−3.36, *P*=0.001).

The multiple linear regression also indicated that the association between CLTI and total cholesterol was no longer significant after adjusting for diabetes (β=−0.179, t=− 1.85, *P*=0.067).

No differences were found in patients with CLTI, between Fontaine stages III and IV, on the serum level of acute‐phase proteins (Table 3).

**Table 3:** Positive (CRP and Fibrinogen) and Negative (HDL, Albumin, and Total Cholesterol) Acute‐Phase Proteins Determined in Patients With CLTI (Fontaine III vs Fontaine IV) at Admission

	Fontaine IV (n=35)		Fontaine III (n=19)		*P* value
	Median	25th–75th percentile	Median	25th–75th percentile	
CRP, g/dL	38.15	2.90–53.28	10.71	4.90–57.13	0.714
Fibrinogen, mg/dL	491.69	312.00–615.75	327.58	253.25–588.75	0.373
HDL, mg/dL	38.90	30.00–45.50	50.07	30.00–43.00	0.730
Albumin, g/dL	3.39	3.10–4.00	4.05	3.30–4.65	0.051
Total cholesterol, mg/dL	143.52±95 (130.18–154.96)		152.80±95 (129.56–176.04)		0.442

CLTI indicates chronic limb‐threatening ischemia; CRP, C‐reactive protein; and HDL, high‐density lipoprotein.

### Evolution of Inflammatory Parameters

**Figure 1:**
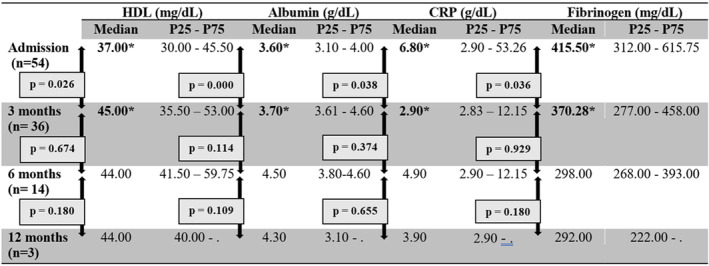
Evolution of negative acute‐phase proteins (HDL, albumin) and positive acute‐phase proteins (CRP and fibrinogen) in patients with CLTI at 3, 6, and 12 months. CLTI indicates chronic limb‐threatening ischemia; CRP, C‐reactive protein; and HDL, high‐density lipoprotein; *P<0.005.

**Figure 2:**
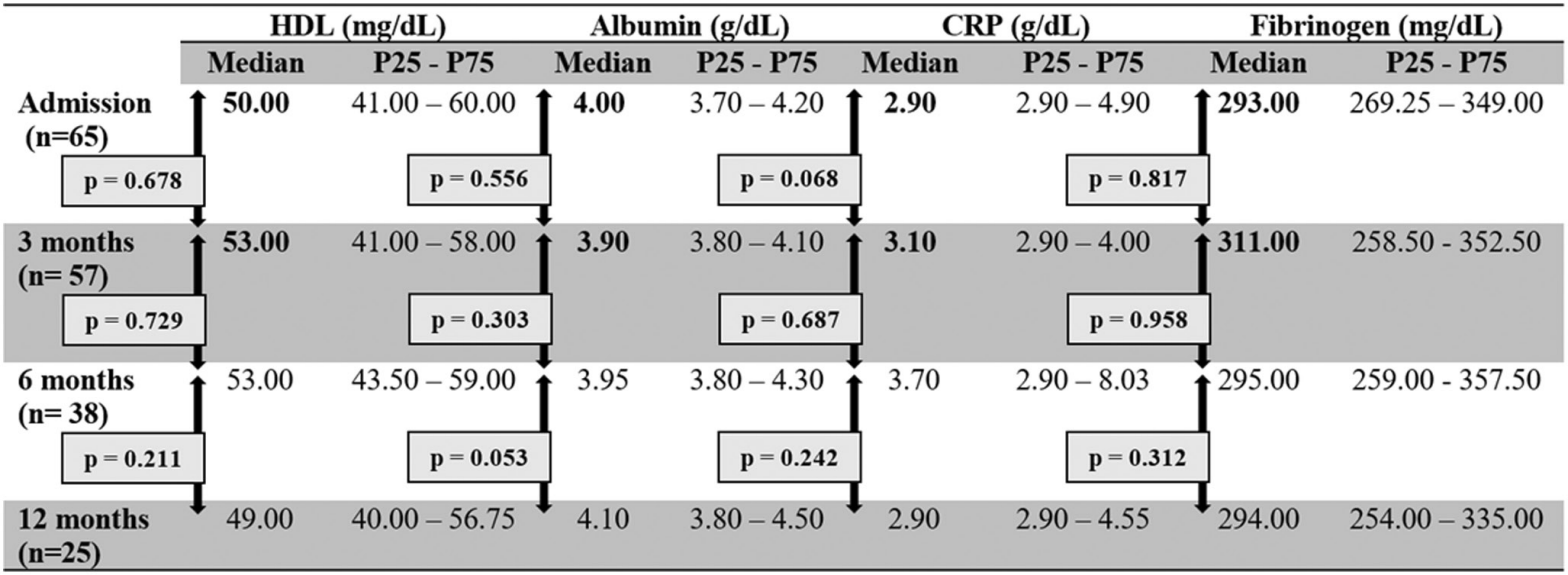
Evolution of negative acute‐phase proteins (HDL and albumin) and positive acute‐phase proteins (CRP and fibrinogen) in patients with claudication at 3, 6, and 12 months. CRP indicates C‐reactive protein; and HDL, high‐density lipoprotein; *P<0.005.

Analyzing the evolution of patients with acute‐phase proteins, we noted that 3 months after the resolution of the ischemic process in patients with CLTI (by amputation or revascularization), there was an increase in serum levels of HDL and albumin (negative acute‐phase proteins) and decrease in CRP and fibrinogen (positive acute‐phase proteins) (Figure 1). These differences were not registered at 6 or 12 months (Figure 1). No differences were noted through time in patients with claudication (Figure 2).

**Figure 3:**
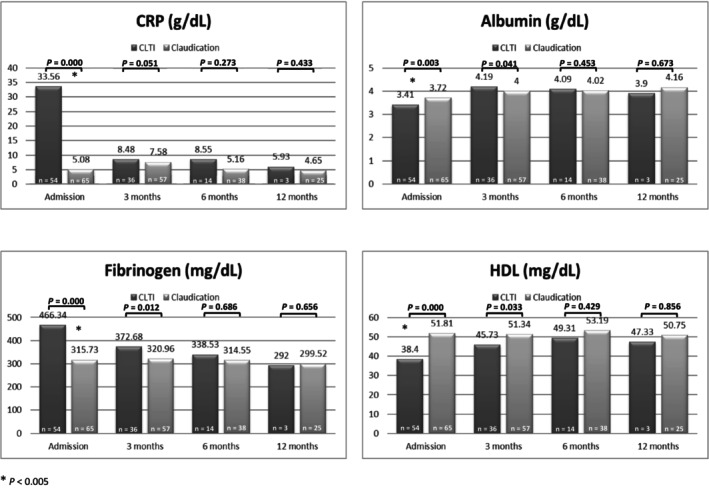
Comparison of acute‐phase proteins between patients with claudication and patients with CLTI at 3, 6, and 12 months. CLTI indicates chronic limb‐threatening ischemia; CRP, C‐reactive protein; and HDL, high‐density lipoprotein.

We also noted that, for most inflammatory markers, the difference between those who had claudication and patients with CLTI was present at admission but disappeared at 6 and 12 months (Figure 3).

## Discussion

To the best of our knowledge, this article is one of the few that reports the possibility of partially reversing the inflammatory state in patients with CLTI after resolution of the CLTI state. The novelty of this study lies in the observation that 3 months following the resolution of the ischemic process, there is a reversal of the inflammatory markers, and the differences between CLTI and claudicants disappear at 6 months. To our knowledge, no other study has compared the inflammatory markers between CLTI and claudicants over such an extended period of time.

### Inflammatory Parameters in CLTI and Patients With Claudication

We observed that patients with CLTI have a significantly higher level in CRP and fibrinogen (positive acute‐phase proteins) and a significantly lower serum level of albumin, and HDL (negative acute‐phase proteins) compared with patients with claudication, as we have previously demonstrated.^9^

We hypothesized that this inflammatory pattern could be caused by the extension of atherosclerosis lesions, tissue infection, or the ischemic tissue process. It has been reported that the severe reduction in tissue oxygenation present in patients with CLTI can trigger an inflammatory reaction, leading to an increase in fibrinogen levels.^12^ The elevated fibrinogen levels result in higher blood viscosity, promoting thrombosis, and potentially explaining the higher risk of graft occlusion, stroke, myocardial infarction, or death observed in patients with CLTI.^3^, ^12^ Increased plasma viscosity also impairs the vessel perfusion, particularly in small vessels such as capillaries, increasing tissue ischemia.^13^

The inflammatory reaction also contributes to the generation of a proatherogenic lipid profile characterized by low HDL, oxidized low‐density lipoprotein, and high triglycerides.^14^ We observed that the patients with CLTI had low HDL and high triglyceride levels. Additionally, patients with CLTI had a lower albumin level, which is considered a powerful predictor of death.^14^

Inflammation is also a stimulus for arteriogenesis, the process involving the formation of new collateral arteries, a phenomenon that is common among patients with PAD.^15^ Inflammatory cells play a pivotal role in providing a source of angiogenic and arteriogenic factors.^16^ These factors encompass vascular endothelial growth factor, fibroblast growth factor, platelet‐derived growth factor, and transforming growth factor‐beta.^16^ A study carried out on patients with PAD provided evidence that the levels of angiogenic factors, particularly vascular endothelial growth factor and hepatocyte growth factor, escalates with the severity of limb ischemia.^17^ Among these factors, vascular endothelial growth factor is one of the most extensively studied, playing a fundamental role in endothelial proliferation, migration, and lumen formation.^15^ Research projects have been conducted to use the angiogenic factors to improve the limb salvage.^18^

To investigate whether the inflammation in patients with CLTI is primarily caused by tissue infection, we compared the serum levels of acute‐phase proteins between patients in Fontaine stage III and stage IV, but no differences were found, as suggested by other authors.^13^

Our hypothesis was that the differences in inflammatory markers between CLTI and those with claudication could be explained by the extension and activity of the atherosclerotic process in patients with critical limb ischemia. It is plausible that the atherosclerotic plaques in these patients, when compared with patients with claudication, yield a higher proportion of proinflammatory M1 macrophages compared with the anti‐inflammatory M2 macrophages.^19^ The M1 macrophages produce proinflammatory cytokines such as interleukin‐1β, interleukin‐6, and interleukin‐12, whereas the M2‐activated macrophages contribute to inflammation reduction, resulting in an elevated concentration of anti‐inflammatory cytokines interleukin‐4 and interleukin‐13.^20^ It is established that progressive plaques primarily exhibit M1 macrophage phenotypes and that M2 macrophages predominate in stable plaques, as they produce profibrotic factors.^20^ This microscopic inflammatory pattern within atherosclerotic plaque could potentially play a role in the progression of claudication through CLTI.

Nevertheless, an alternate explanation is conceivable. The pattern of evolution of the inflammatory markers (discussed below) suggests that it is the ischemic tissue process, rather than the extension of atherosclerotic plaques, that causes the differences between CLTI and claudicants.

### Evolution of Inflammatory Parameters

We observed that 3 months after the resolution of the CLTI state (by amputation or surgery), there was a reversion, at least in part, of the inflammatory parameters, with an increase in the negative acute‐phase proteins (HDL and albumin) and a decrease in positive acute‐phase proteins (CRP and fibrinogen). These differences were not observed in patients with claudication or at other periods of time in patients with CLTI. At 3 months, patients with CLTI maintained a higher serum level of CRP and fibrinogen and lower levels of albumin compared with patients with claudication. However, these differences disappeared at 6 months. Our findings align with 2 previous studies: Bismuth et al reported a decrease in CRP and an increase in HDL and albumin after revascularization in 30 patients with CLTI.^21^ Woodburn et al compared fibrinogen levels in 56 patients with CLTI at admission and 16 weeks after revascularization, observing a reduction in fibrinogen levels, although they remained higher than those of the controls.^3^ The normalization of fibrinogen levels following successful vascular surgery of critically ischemic limbs through revascularization (either surgical or endovascular) or through amputation suggests that tissue ischemia may stimulate hepatic fibrinogen synthesis, possibly through interleukin‐6 produced by activated monocytes.^12^ Both of these studies analyzed the data at 3 months, and no other study has examined the data over a longer follow‐up period.

### Clinical Implications

Demonstrating that the resolution of CLTI can decrease for >3 months, the serum levels of inflammatory markers suggest that the deleterious impact of inflammation can be mitigated with a prompt intervention.

This study also highlights the potential role of anti‐inflammatory medical therapy in patients with PAD. For example, canakinumab is an interleukin‐1β inhibitor that improved walking performance in patients with claudication while reducing blood CRP and interleukin‐6.^22^ Statins have been associated with a reduction in systemic inflammation, as measured by CRP, and have shown benefits in terms of a lower mortality rate, reduced major adverse cardiovascular and cerebrovascular events, and longer amputation‐free survival in patients with CLTI.^6^, ^22^, ^23^ The benefit of statins is closely related to their anti‐inflammatory effect.^24^ However, statins also exhibit a procalcific effect, leading to an increase in calcium density.^25^ The long‐term use of statins was associated with severity of coronary artery calcium score, as emphasized in a recent paper.^26^ It is widely recognized that patients with CLTI exhibit severe calcification across nearly all arterial territories, a factor that can significantly contribute to the unfavorable outcomes associated with this disease.^27^ Statins may have a role in this process, which was not yet explored in the specific group of patients with CLTI. Fibrates also exhibit fibrinogen‐lowering action.^6^

This paper has several strengths:

Comprehensive investigation: The paper explores the association between PAD and inflammation and the evolution of inflammatory parameters in patients with claudication and those with CLTI over a period of 3, 6, and 12 months.

Comparison between patients with claudication and patients with CLTI over an extended period of time: The study compares patients with claudication and patients with CLTI in terms of their inflammatory parameters. By examining the differences between these 2 groups, the researchers can better understand the impact of disease severity on inflammation.

Findings on the reversal of the inflammatory state in patients with CLTI: The study reports that 3 months after the resolution of the ischemic process, there is a partial reversal of the inflammatory state in patients with CLTI. This finding suggests that prompt intervention and anti‐inflammatory therapies may help alleviate the detrimental effects of inflammation in patients with PAD.

Comparing stages III and IV of the Fontaine classification: The article reports no difference in the serum levels of acute‐phase proteins between patients in stage III and those in stage IV. This fact underlines the role of the ischemic tissue in the inflammatory changes registered in patients with CLTI.However, this research work also has limitations:

Sample size: The study included a total of 119 patients followed by the main author, which may not be representative of the entire PAD population followed at our institution. A larger sample size could provide more robust and generalizable results.

Prevalence of men in the studied population: Approximately 80% of the recruited sample were men. Therefore, the results cannot be generalized to all populations.

Short follow‐up period (even if the longest recorded in the literature): The study evaluated the evolution of inflammatory parameters at 3, 6, and 12 months. However, a longer follow‐up period could provide more insights into the long‐term changes and trends in inflammatory markers. However, as far as we know, this is the longest follow‐up published.

Single‐center study: The study was conducted at a single institution, which may limit the generalizability of the findings. A multicenter study could provide a more comprehensive understanding of the topic.

Losses during the follow‐up: 78.15% of patients attended the first 2 appointments, and 43.70% attended the third appointment. Only 23.53% were observed until the end of the study. Although the main results of our study (differences between patients with claudication and CLTI and reversion of inflammatory parameters) were registered at the first and second appointments, these losses are a weak point of this work. The withdrawal can be explained by the characteristics of patients with PAD (frailty, older, and dependent on their relatives) and by the fact that this research started in January 2018, and midway through the study, we faced the COVID‐19 pandemic.

Other factors: The article did not investigate other potential factors that could influence the inflammatory state, such as genetics and the practice of exercise and diet.

Causality: This research work is also not able to establish a causal relationship between the resolution of the ischemia process and the improvement in the inflammatory data.

## Conclusions

This study provides valuable insights into the evolution of inflammatory parameters in patients with PAD, specifically comparing those with CLTI with those with claudication. It emphasizes that patients with CLTI demonstrated heightened serum levels of positive acute‐phase proteins and reduced levels of negative acute‐phase proteins, indicating an inflammatory state. This condition can be at least partially reverted after the resolution of the ischemic process, thus potentially mitigating the negative impact of inflammation.

## Sources of Funding

This work was supported by the Portuguese Society of Vascular Surgery. This work was developed under the scope of project NORTE‐01‐0145‐FEDER‐000013, supported by the Northern Portugal Regional Operational Programme (NORTE 2020) under the Portugal Partnership Agreement, through the European Regional Development Fund (FEDER), and by National funds, through the Foundation for Science and Technology (FCT), project UIDB/50026/2020 and UIDP/50026/2020.

## Disclosures

None.
